# The function of chloroplast ferredoxin‐NADP^+^ oxidoreductase positively regulates the accumulation of bamboo mosaic virus in *Nicotiana benthamiana*

**DOI:** 10.1111/mpp.13174

**Published:** 2021-12-17

**Authors:** I‐Hsuan Chen, Xiang‐Yu Chen, Guan‐Zhi Chiu, Ying‐Ping Huang, Yau‐Heiu Hsu, Ching‐Hsiu Tsai

**Affiliations:** ^1^ Graduate Institute of Biotechnology National Chung Hsing University Taichung Taiwan; ^2^ Advaced Plant Biotechnology Center National Chung Hsing University Taichung Taiwan

**Keywords:** BaMV, chloroplast, electron transport chain, ferredoxin NADP^+^ oxidoreductase, in vitro replication assay photosynthesis enzyme, viral RNA accumulation

## Abstract

A gene down‐regulated in *Nicotiana benthamiana* after bamboo mosaic virus (BaMV) infection had high identity to the nuclear‐encoded chloroplast ferredoxin NADP^+^ oxidoreductase gene (*NbFNR*). NbFNR is a flavoenzyme involved in the photosynthesis electron transport chain, catalysing the conversion of NADP^+^ into NADPH. To investigate whether NbFNR is involved in BaMV infection, we used virus‐induced gene silencing to reduce the expression of *NbFNR* in leaves and protoplasts. After BaMV inoculation, the accumulation of BaMV coat protein and RNA was significantly reduced. The transient expression of NbFNR fused with orange fluorescent protein (OFP) localized in the chloroplasts and elevated the level of BaMV coat protein. These results suggest that NbFNR could play a positive role in regulating BaMV accumulation. Expressing a mutant that failed to translocate to the chloroplast did not assist in BaMV accumulation. Another mutant with a catalytic site mutation could support BaMV accumulation to some extent, but accumulation was significantly lower than that of the wild type. In an in vitro replication assay, the replicase complex with FNR inhibitor, heparin, the RdRp activity was reduced. Furthermore, BaMV replicase was revealed to interact with NbFNR in yeast two‐hybrid and co‐immunoprecipitation experiments. Overall, these results suggest that NbFNR localized in the chloroplast with functional activity could efficiently assist BaMV accumulation.

## Graphical abstract

The nuclear‐encoded chloroplast ferredoxin NADP^+^ oxidoreductase gene (NbFNR) could assist bamboo mosaic virus accumulation efficiently when expressed in *Nicotiana benthamiana* leaves but not the mutants with failure in translocating to the chloroplast (NbFNR/ΔTP) and with a catalytic site mutation (NbFNR/mut).

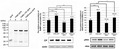

## 1 INTRODUCTION

Bamboo mosaic virus (BaMV), a flexuous rod virus of approximately 490 × 15 nm, causes mosaic symptoms on bamboo plants. BaMV has a single‐stranded positive‐sense RNA genome of about 6400 nucleotides with a 5′ cap structure (m^7^GpppG) and a 3′ poly(A) tail; *Bamboo mosaic virus* belongs to the genus *Potexvirus* of the family *Alphaflexiviridae* (Lin et al., 1992). The 3′ untranslated region (3′ UTR) of BaMV RNA forms a tertiary structure (Cheng & Tsai, 1999) and has roles in minus‐strand RNA synthesis, polyadenylation, and long‐distance movement (Chen, Huang, et al., 2017).

The BaMV genome has five open reading frames (ORFs) (Lin et al., 1994). ORF 1 encodes a 155‐kDa replicase involved in viral RNA replication with AdoMet‐dependent guanylyltransferase activity (Huang et al., 2004; Li, Chen, et al., 2001), RNA 5′‐triphosphatase and NTPase activity (Li, Shih, et al., 2001), and RNA‐dependent RNA polymerase (RdRp) activity (Li et al., 1998). Overlapping ORFs 2 to 4 encode movement proteins of 28, 13, and 6 kDa, respectively (Lin et al., 2004, 2006). ORF 5 encodes a 25‐kDa viral capsid protein (CP) for viral encapsidation, movement, and symptom development in plants (Hung, Hu, et al., 2014; Hung, Huang, et al., 2014; Lan et al., 2010; Lee et al., 2011).

Every virus needs cellular host factors to complete its successful infection. Thus, host factors are critical for most steps of virus infection, especially single‐stranded positive‐sense RNA viruses (Medina‐Puche & Lozano‐Duran, 2019; Whitham & Wang, 2004). Once an RNA virus enters a host cell, it needs the host translation machinery for viral protein synthesis (Nicholson & White, 2011; Thivierge et al., 2005). These viral proteins, such as RdRp, recruit host factors to form a viral replication complex (Ahlquist et al., 2003; Ishibashi & Ishikawa, 2016; Laliberte & Sanfacon, 2010; Noueiry et al., 2003). Following replication, more host factors are needed for the viral RNA complex to move to adjacent cells, including intracellular tracking and cell‐to‐cell movement, for further infection (Benitez‐Alfonso et al., 2010; Cheng, 2017; Liou et al., 2015; Lucas, 2006; Niehl & Heinlein, 2011; Schoelz et al., 2011).

When BaMV infects its host plants, the viral RNA has to recruit host proteins to benefit all steps of the infection cycle and respond to all challenges from the host (Huang et al., 2017). Consequently, the gene expression profile of the infected host fluctuates. Therefore, studying up‐ or down‐regulated host proteins could help to reveal the mechanism of viral infection. We used cDNA amplified fragment length polymorphism (AFLP) to isolate the differentially expressed genes in *Nicotiana benthamiana* plants after BaMV infection (Cheng et al., 2010). One of the down‐regulated genes, *ACAG1*, was found to participate in BaMV infection and shows homology to the partial sequence of ferredoxin‐NADP^+^‐oxidoreductase (FNR) in *N. benthamiana*.

FNR is a nuclear‐encoded plastid flavoenzyme of approximately 35 kDa and is localized at the thylakoid membrane. At the end of the photosynthetic electron transport chain, the energized electrons are transferred from the photosystem I protein complex (PSI) to ferredoxin (Fd) (Chitnis, 2001; Haehnel, 1984), then further transferred from Fd to NADP^+^ (Carrillo & Ceccarelli, 2003; Chitnis, 2001). The final products, NADPH and ATP, can be used for neutralizing oxidation stress and for carbon fixation in the Calvin cycle, respectively (Arakaki et al., 1997; Medina, 2009; Morigasaki et al., 1993). The reaction catalysed by FNR is as follows: 2Fd (Fe^+2^) + NADP^+^ + H^+^ ⇄ 2Fd (Fe^+3^) + NADPH (Mulo, 2011).

In this study, we examined the function of the nuclear‐encoded chloroplast FNR of *N. benthamiana* and found it was involved in regulating BaMV accumulation. NbFNR mutants unable to be transported to the chloroplast, or lacking functional activity in electron transport, could not efficiently support BaMV accumulation.

## 2 RESULTS

### 2.1 *NbFNR* down‐regulation results in chlorosis of *N. benthamiana* and decreases the accumulation of BaMV RNA

In a previous study, we used cDNA‐AFLP to screen differentially expressed genes in BaMV‐inoculated *N. benthamiana* plants (Cheng et al., 2010). One of the down‐regulated genes, *ACAG1*, is a homolog of ferredoxin‐NADP^+^ oxidoreductase (FNR) and has 94% amino acid identity sequence with *Nicotiana tabacum* (GenBank: O04977.1) (Figure S1); therefore, we designated this down‐regulated gene *NbFNR* (GenBank: MH511674.1).

**FIGURE 1:**
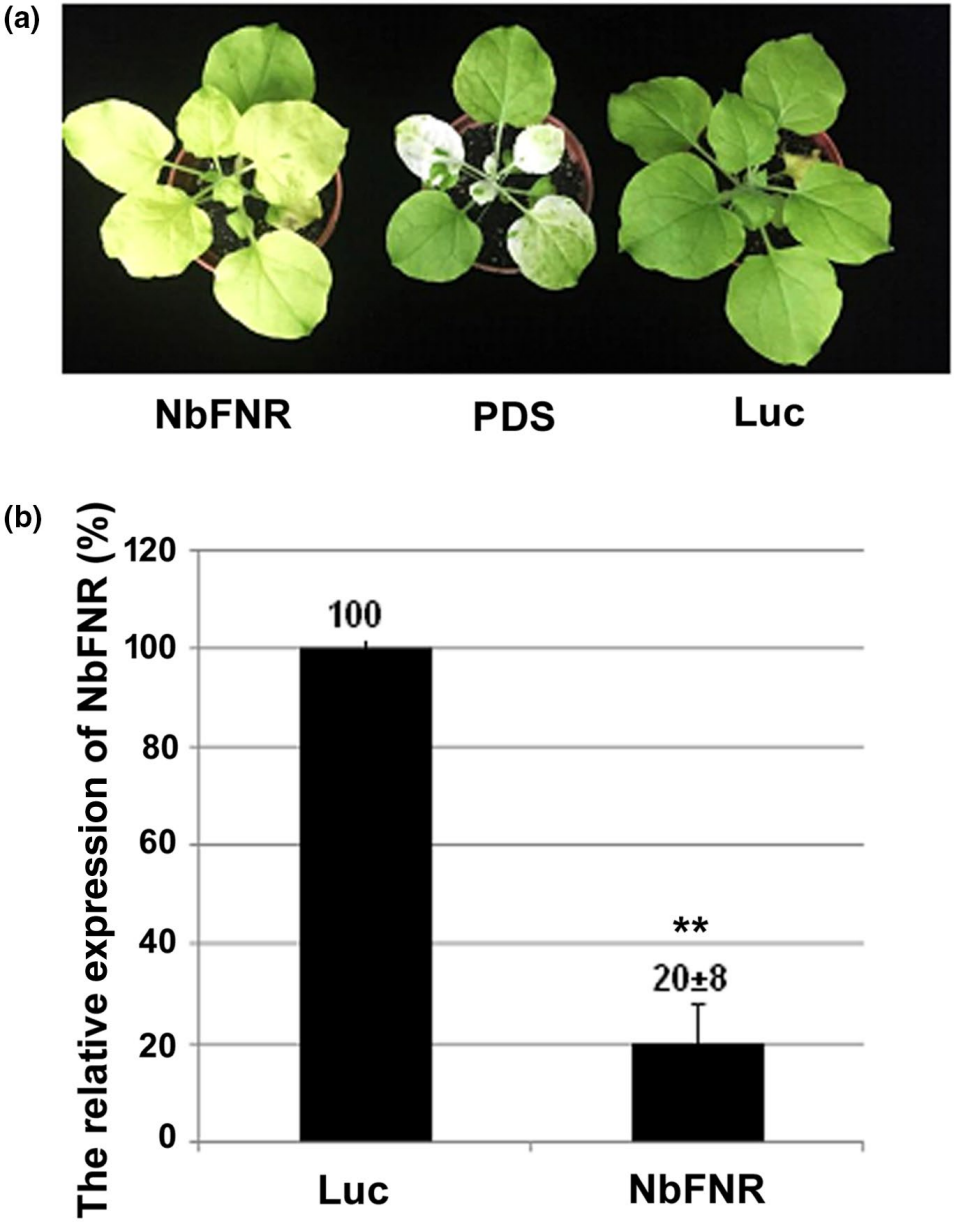
Phenotype of *NbFNR*‐knockdown plants and knockdown efficiency. (a) The phenotype of knockdown plants. *Phytoene desaturase* (PDS) knockdown was a positive control and plants showed a photobleaching phenotype. *Luciferase* (Luc) knockdown was a negative control. *NbFNR*‐knockdown plants displayed a uniform pale chlorosis phenotype. (b) The relative expression of *NbFNR* in *Luc*‐ and *NbFNR*‐knockdown plants was measured by reverse transcription quantitative PCR. The expression of *actin* gene was used for normalization. Data above bars are mean ± *SE*. Asterisks indicate statistically significance difference by Student's *t* test (**p < 0.01). All samples were run at least three times

To reveal the role of *NbFNR* in BaMV infection, we used tobacco rattle virus (TRV)‐based virus‐induced gene silencing (VIGS) to knock down the expression of *NbFNR* in *N. benthamiana* plants. Reducing the expression of *NbFNR* caused a chlorosis phenotype (Figure 1a). The expression of *NbFNR* in the knockdown plants was 20% of that of control plants (Figure 1b).

**FIGURE 2:**
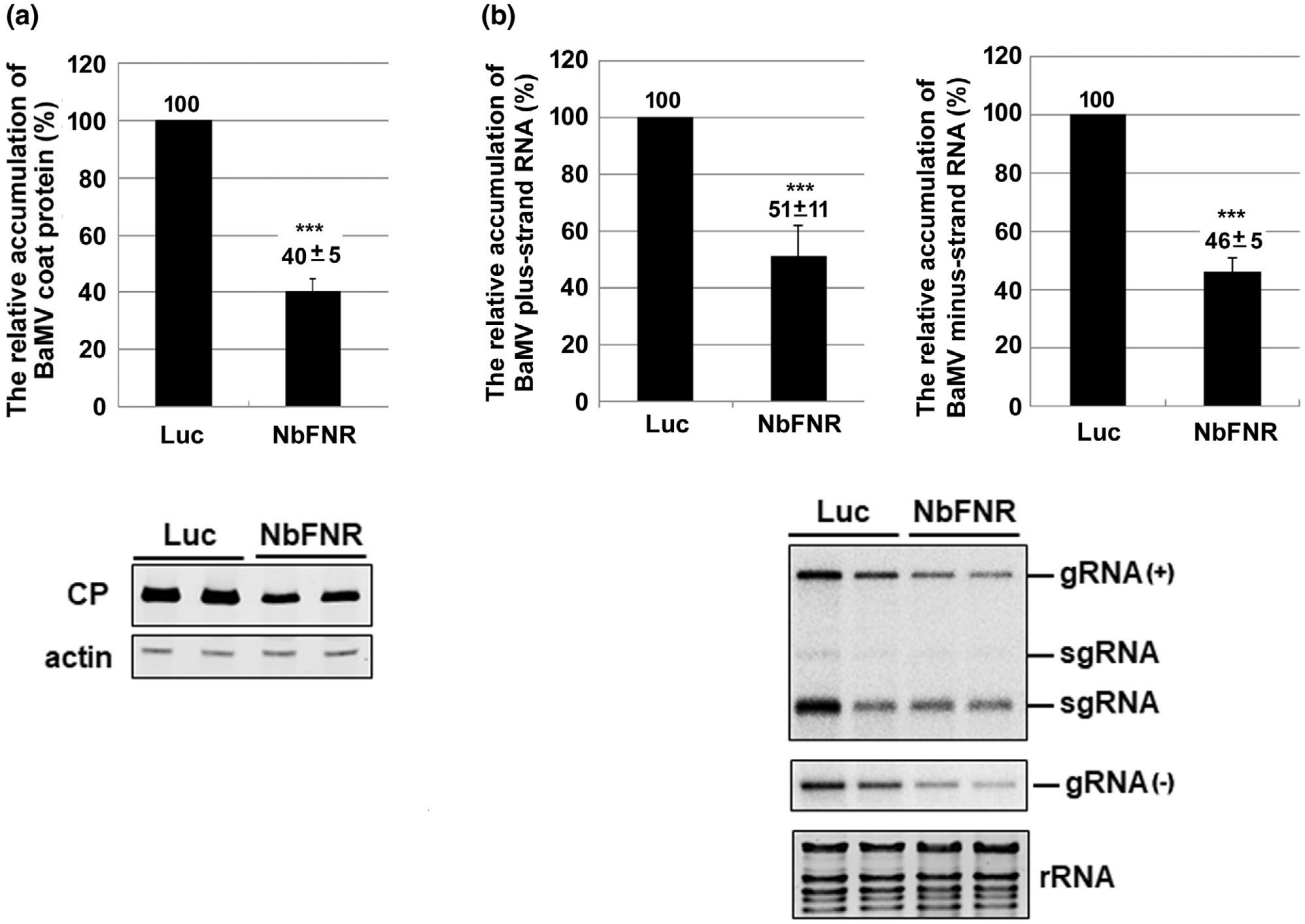
Relative accumulation of BaMV coat protein (CP) and RNA in *NbFNR*‐knockdown plants. (a) Western blot analysis of the accumulation of BaMV coat protein. Actin was a loading control for normalization. (b) Northern blot analysis was used to determine the accumulation of BaMV genomic RNA and anti‐genomic RNA, indicated as gRNA (+) and gRNA(−), respectively. The viral subgenomic RNA is indicated as sgRNA. The accumulation of BaMV in Luc (*luciferase*‐knockdown control) was set to 100% for comparison. Data above bars are mean ± *SE* analysed from at least three independent experiments. Asterisks indicate statistically significance differences by Student's *t* test (***p < 0.001). A representative blot is shown in the figure

Because *NbFNR*‐knockdown plants showed a chlorosis phenotype, the expression of the large subunit of RuBisCO (rbcL) was affected (Figure S2). Using rbcL as the loading control for normalization might lead to a misjudgment of the relative accumulation of viral product (Figure S2). Therefore, the expression of actin was used as the loading control for the western blot analysis. The accumulation of BaMV coat protein in *NbFNR*‐knockdown *N. benthamiana* leaves was approximately 40% of that of control leaves (Figure 2a). The accumulation of BaMV genomic RNA (the plus‐strand) and anti‐genomic RNA (the minus‐strand) in *NbFNR*‐knockdown leaves was approximately 51% and 46%, respectively, of that of control leaves (Figure 2b).

### 2.2 Accumulation of BaMV is less efficient in *NbFNR*‐knockdown protoplasts

**FIGURE 3:**
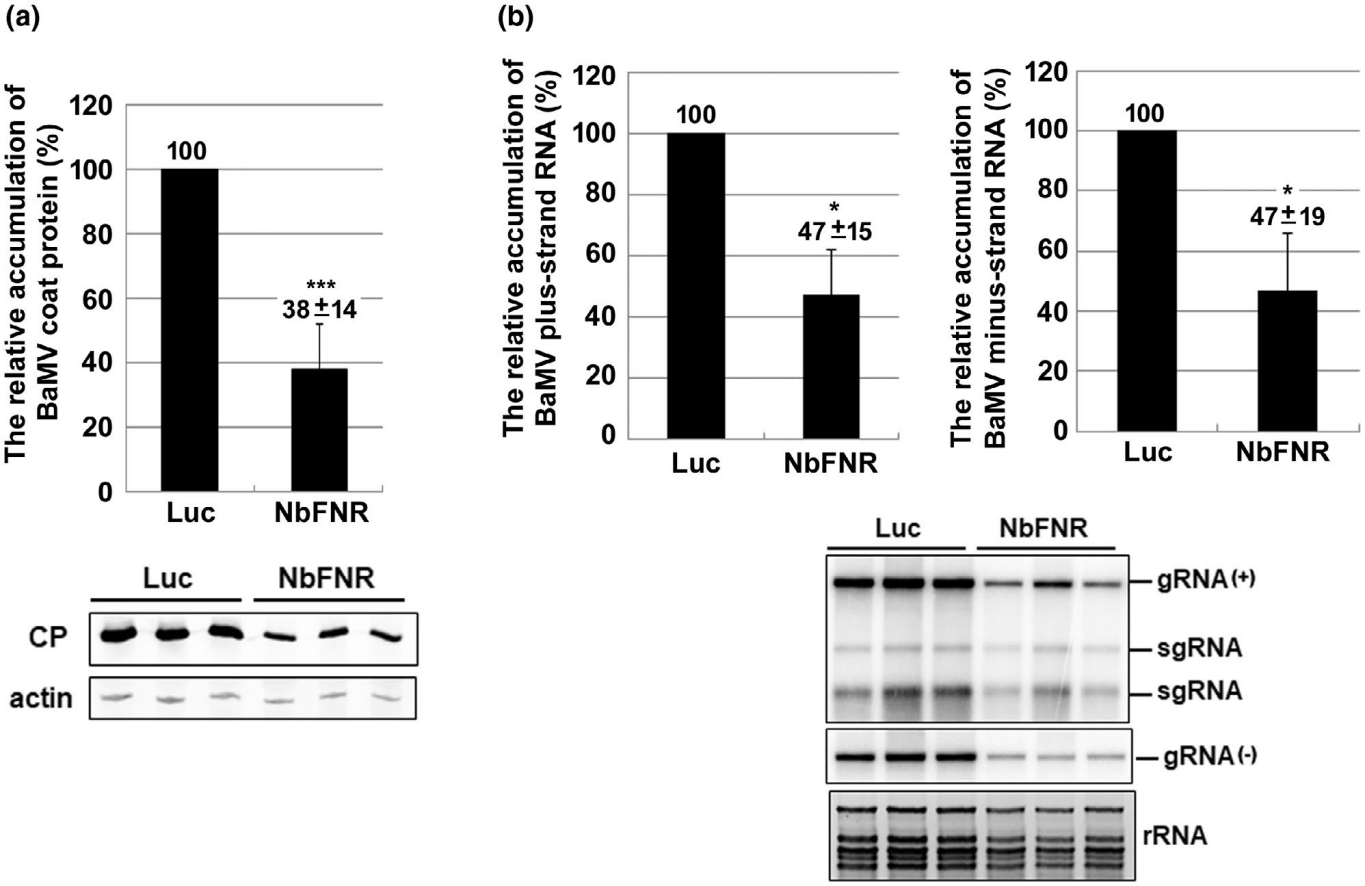
Relative accumulation of BaMV in *NbFNR*‐knockdown protoplasts. Total protein and RNA were extracted from the control (*Luc*‐knockdown protoplasts) and *NbFNR*‐knockdown protoplasts at 24 h postinoculation with 500 ng of BaMV RNA. (a) Western blot analysis of the accumulation of BaMV coat protein (CP). Actin was a loading control for normalization. (b) Northern blot analysis was used to determine the accumulation of BaMV genomic RNA and anti‐genomic RNA, indicated as gRNA (+) and gRNA(−), respectively. The subgenomic RNAs (sgRNA) were not quantified. Data above bars are mean ± *SE* analysed from three independent experiments. The accumulation of BaMV in *Luc*‐knockdown protoplasts was set to 100% for comparison. Asterisks indicate statistically significant differences by Student's *t* test (*p < 0.05, ***p < 0.001). Luc, *luciferase*‐knockdown protoplasts; NbFNR, *NbFNR*‐knockdown protoplasts. A representative blot is shown in the figure

The reduction of BaMV RNA in *NbFNR*‐knockdown leaves could result from effects on viral RNA replication or cell‐to‐cell movement. To clarify the possible effects of *NbFNR* in BaMV accumulation, *NbFNR*‐knockdown protoplasts were prepared for viral RNA inoculation. Protoplasts were isolated from *NbFNR*‐knockdown leaves with the cell wall removed to exclude the involvement of viral movement. Western blot analysis revealed the BaMV coat protein level in knockdown protoplasts decreased to 38% of that of control protoplasts at 24 h postinoculation (hpi) (Figure 3a). Northern blot analysis revealed the BaMV genomic and anti‐genomic RNA levels decreased to 47% of those of control protoplasts at 24 hpi (Figure 3b). These results suggest that down‐regulating *NbFNR* expression could diminish the accumulation of BaMV at the single‐cell level.

### 2.3 BaMV accumulation is increased in *NbFNR*‐expressing leaves

**FIGURE 4:**
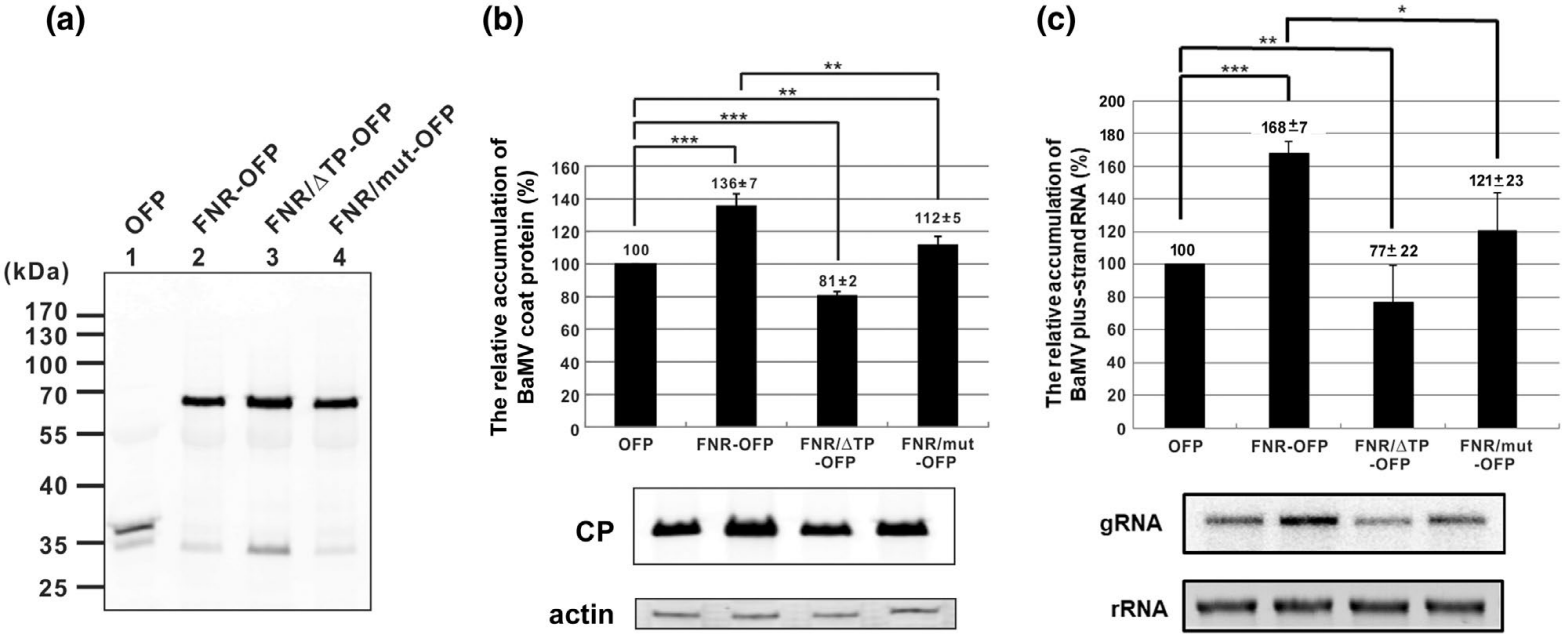
Accumulation of BaMV in plants transiently expressing NbFNR and its derivatives following agroinfiltration. (a) Western blot analysis of the expression of pEpyon vector (orange fluorescent protein [OFP] only), NbFNR*‐*OFP, and its derivatives in transiently expressing plants. (b) and (c) Western and northern blot analysis were used to determine the accumulation of BaMV coat protein (CP) and viral genomic RNA (gRNA), respectively, in plants expressing *NbFNR* or its derivatives. Total protein or RNA was extracted from plants agroinfiltrated with the pEpyon vector (OFP only) and NbFNR*‐*OFP constructs at 3 days postinoculation with 200 ng of BaMV. The accumulation of BaMV CP or RNA in OFP‐only expressing plants was set to 100% for comparison. Data above bars are mean ± *SE* obtained from four independent experiments. Asterisks indicate statistically significant differences by Student's *t* test (*p < 0.05, **p < 0.01, ***p < 0.001). FNR/ΔTP‐OFP, FNR with transit peptide removed; FNR/mut‐OFP, FNR with mutations in the catalytic site; β‐actin was used as a loading control

The results of *NbFNR*‐knockdown experiments suggested that *NbFNR* could play a helper role in BaMV accumulation. To provide direct evidence supporting this hypothesis, we transiently expressed NbFNR fused with either orange fluorescent protein (OFP) or a T7 tag at the C‐terminus in *N. benthamiana* leaves and inoculated with BaMV. The expression of NbFNR‐OFP (approximately 70 kDa; Figure 4a) or NbFNR‐T7 (39 kDa) and OFP‐T7 (37 kDa) (Figure S3a) was detected by western blot assay with the antibody against OFP or T7‐tag, respectively. The BaMV coat protein level in NbFNR‐expressing leaves was increased to 136% (NbFNR‐OFP) (Figure 4b) or 137% (NbFNR‐T7) (Figure S3b) of that of control leaves. The genomic RNA was increased to 168% of that of the control (Figure 4c). The results indicated that a C‐terminal fusion of NbFNR with either a small tag (T7) or a 35 kDa‐polypeptide (OFP) could give a similar effect in assisting the accumulation of BaMV. Overall, these results confirmed that NbFNR plays a positive role in assisting BaMV accumulation.

### 2.4 NbFNR is localized in chloroplasts of *N. benthamiana*

**FIGURE 5:**
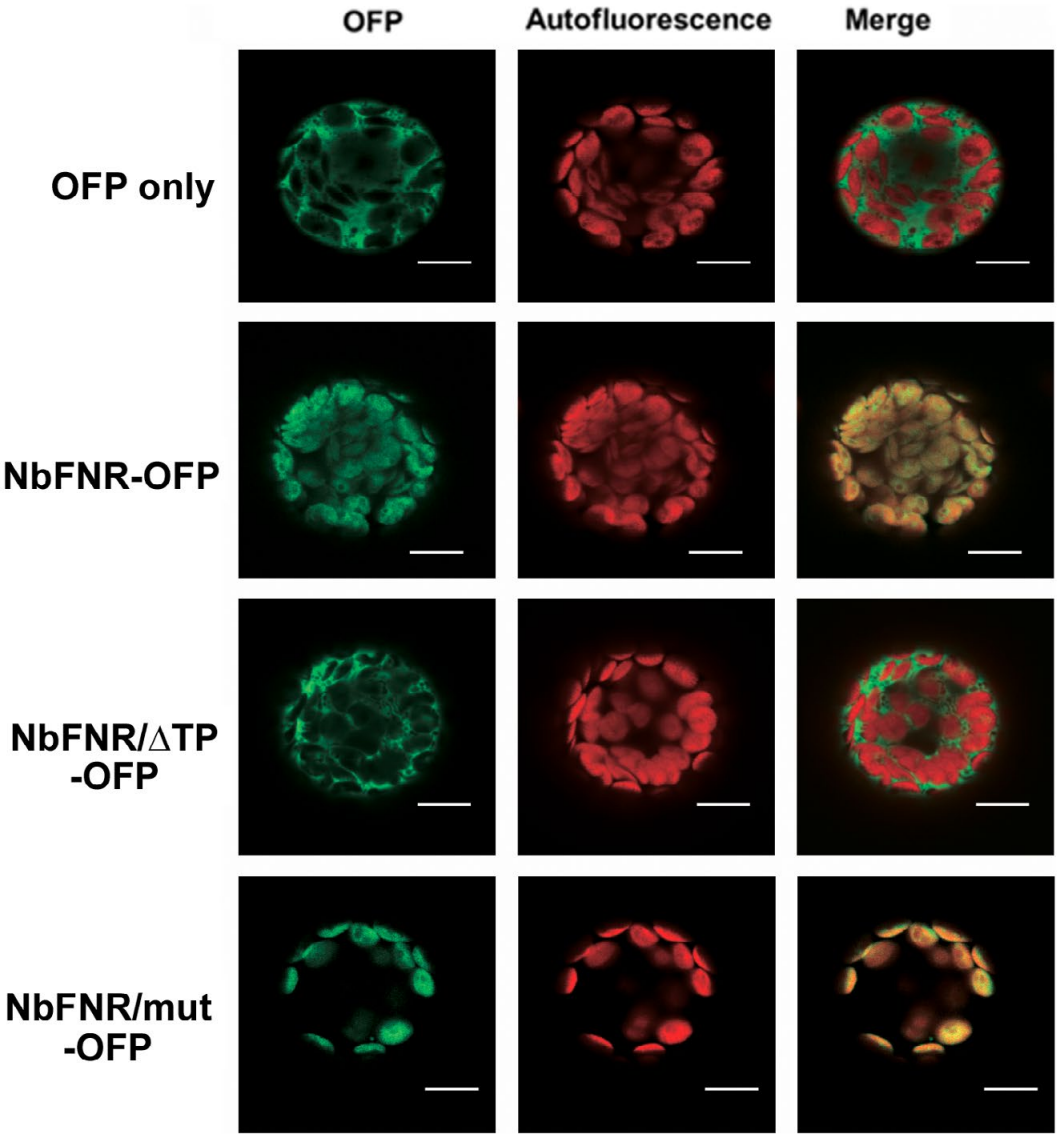
Localization of NbFNR‐OFP in *Nicotiana benthamiana* protoplasts. Confocal microscopy of localization of orange fluorescent protein (OFP) only, NbFNR‐OFP, and its derivatives transiently expressed in *N. benthamiana* protoplasts. OFP is shown in green and the autofluorescence of chloroplasts is in red. Scale bar: 10 μm

To localize NbFNR in plant cells, NbFNR‐OFP was transiently expressed together with the *Potato virus Y* silencing suppressor (HC‐Pro) by agroinfiltration into *N. benthamiana* leaves (Baumberger et al., 2007). On confocal microscopy, the signal of NbFNR‐OFP detected with the orange channel co‐localized with the autofluorescent signals from chloroplasts in both leaves (Figure S4) and protoplasts (Figure 5). The localization of free OFP is shown in Figure 5 but should also be noted here in the Results. Therefore, the NbFNR we isolated and used in this study was confirmed to localize in chloroplasts as expected. Furthermore, the localization in the chloroplast of NbFNR‐OFP showed no obvious relocalization after BaMV infection (Figure S5).

### 2.5 The activity of NbFNR is involved in BaMV accumulation

To test whether the activity of NbFNR is required for BaMV accumulation in chloroplasts, we created two mutants that either could not be transported to chloroplasts (NbFNR/ΔTP) or had reduced catalytic activity with mutations at active sites (S140 and E360) (Dumit et al., 2010) (NbFNR/mut). NbFNR/ΔTP failed to be transported to chloroplasts (Figure 5) and could not assist in BaMV accumulation (Figure 4), which might have a negative effect on BaMV accumulation. The dominant‐negative effect of NbFNR/ΔTP could result from interference with transporting the proteins required for BaMV accumulation into the chloroplasts. The mutant S140A/E360A, affecting the catalytic site, still helped with BaMV accumulation to some extent (112% of that of the control level); however, the wild‐type NbFNR had significantly enhanced accumulation of BaMV as compared with NbFNR/mut (Figure 4).

**FIGURE 6:**
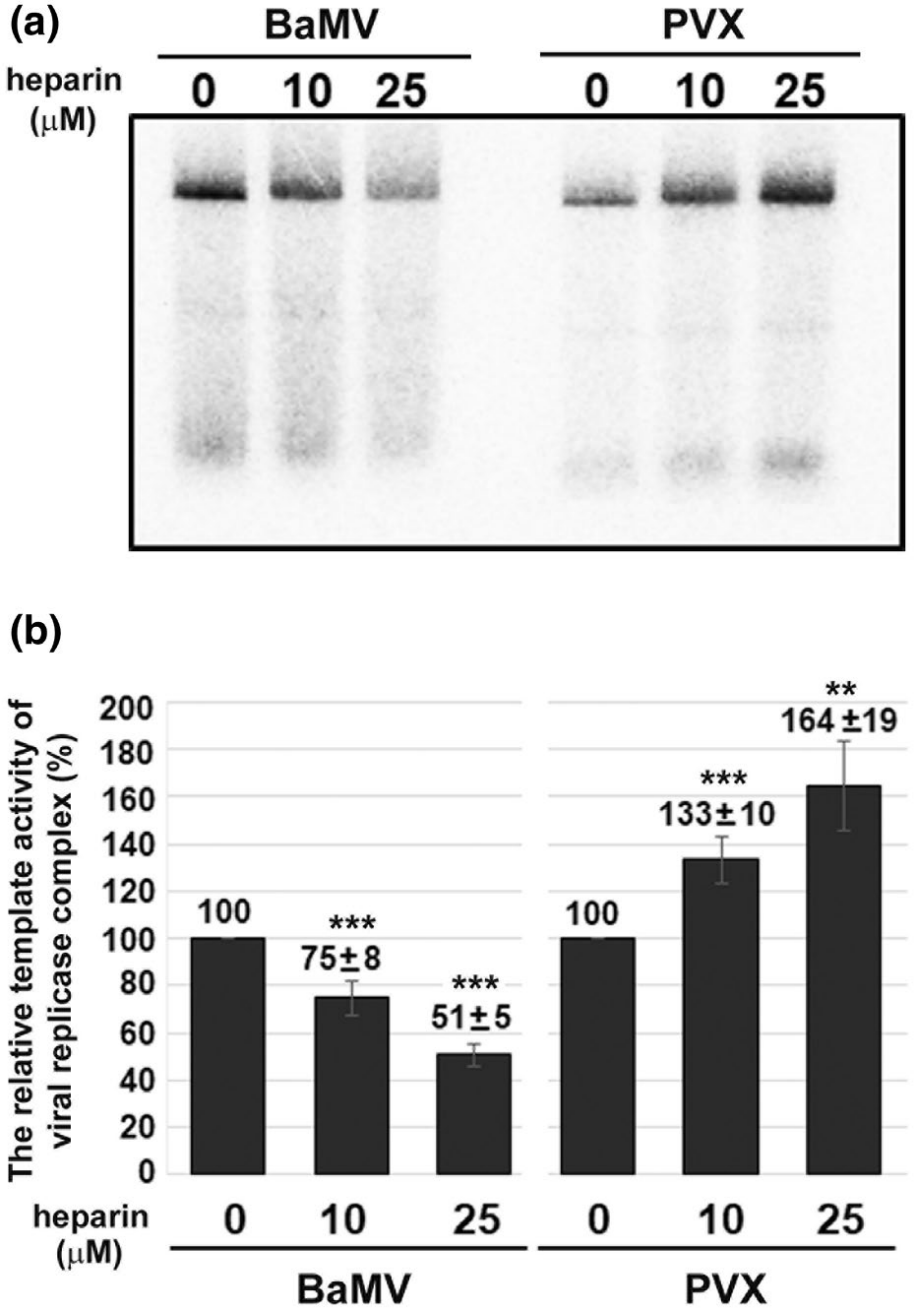
The in vitro replication assays with the replicase complex extracted from BaMV‐ and PVX‐infected *Nicotiana benthamiana* leaves. (a) Representative results of the endogenous template activity in an in vitro replication assay. The concentration of heparin used in the reaction is indicated. (b) The relative template activity of viral replicase complex in the in vitro replication assay. The endogenous template activity without heparin was set to 100%. Each treatment of the reaction is the mean relative activity ± standard error derived from three independent experiments

To confirm the activity of NbFNR is involved in BaMV accumulation, we performed an in vitro replication assay with heparin, an FNR inhibitor (Hosler & Yocum, 1985). The replicase complex was extracted from *N*. *benthamiana* plants infected with BaMV or potato virus X (PVX) (Cheng et al., 2001; Lin et al., 2005). The endogenous template activity of BaMV replicase was reduced in the presence of heparin in a dose‐dependent manner (Figure 6). By contrast, the endogenous template activity of PVX replicase was increased in the presence of heparin in a dose‐dependent manner (Figure 6). It has been reported that the silencing suppressor P25 of PVX can interact with ferredoxin 1 (FD1), which blocks the electron transport in the chloroplast. Thus, the down‐regulation of FD1 expression elevates PVX accumulation (Yang et al., 2020). The inhibition of FNR activity with heparin interfered with binding by both FD and NADP(H), which subsequently blocked the electron transport and enhanced the accumulation of PVX (Figure 6). These results imply that electron transport could play a functional role in anti‐PVX replication. Based on the results derived from catalytic site mutation of NbFNR and the presence of an FNR inhibitor in an in vitro replication assay, we conclude that the activity of FNR could be crucial in assisting efficient BaMV replication.

### 2.6 BaMV replicase interacts with NbFNR

**FIGURE 7:**
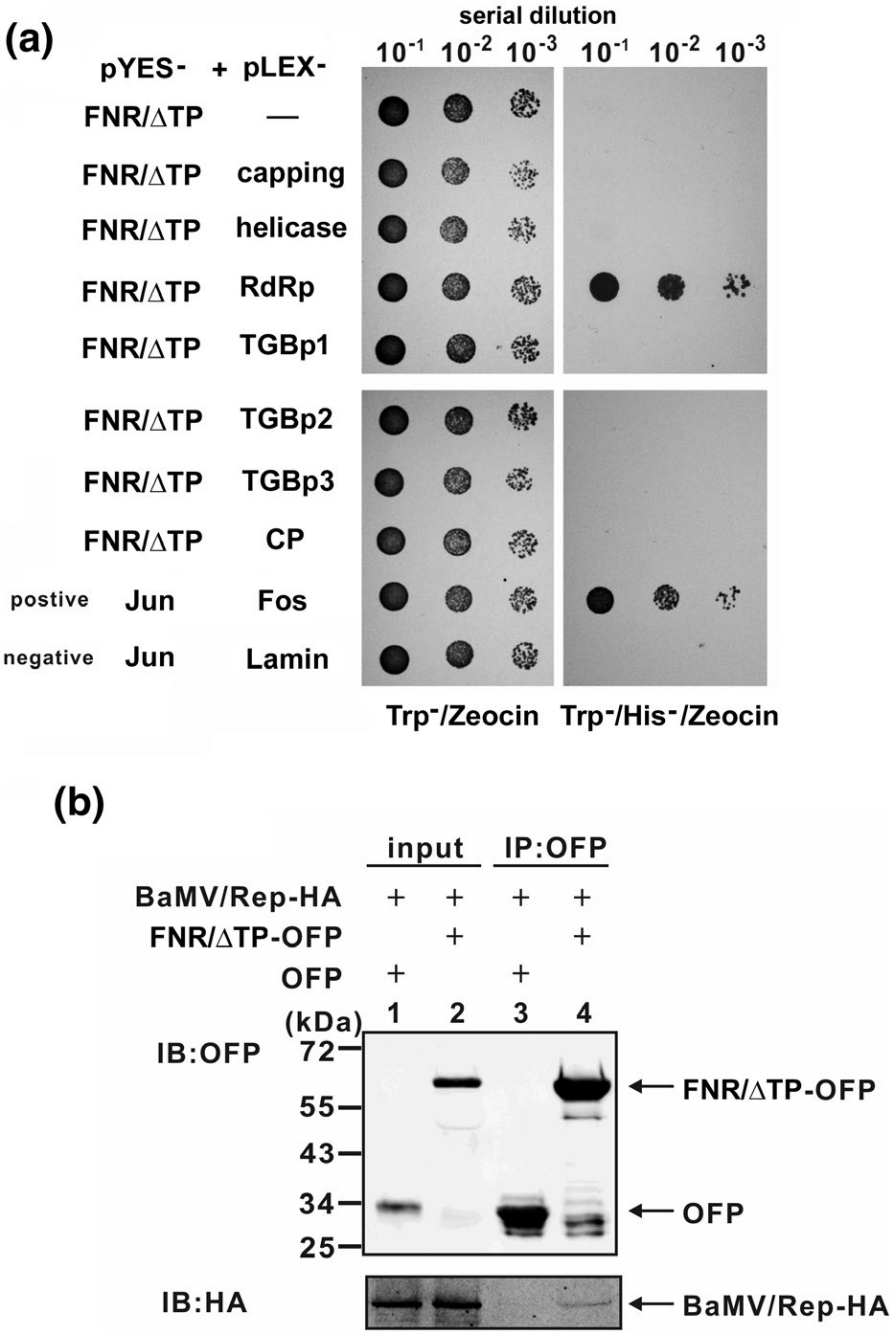
The interaction of NbFNR with BaMV replicase. (a) Interaction of NbFNR/ΔTP (lacking transit pepptide) with BaMV‐encoded polypeptides in yeast cells. Yeast strain L40 co‐transformed with the indicated plasmids was subjected to 10‐fold serial dilution and incubated with minimal medium lacking tryptophan and histidine supplemented with Zeocin to identify protein interactions. Yeast containing pYESTrp‐Jun and pHyLEX/Zeo‐Fos was a positive control; yeast containing the vector pYESTrp‐Jun and pHyLEX/Zeo‐Lamin was a negative control. (b) Total proteins (input) were orange fluorescent protein (OFP) only and OFP‐fused NbFNR/ΔTP as indicated and HA‐tagged BaMV replicase. Total proteins were immunoprecipitated with anti‐OFP beads (IP:OFP) and immunoblotted (IB) with the antibody against OFP or HA‐tag

To examine whether NbFNR interacts with any virus‐encoded protein, we performed a yeast two‐hybrid experiment (Figure 7a). Because transport to the chloroplast is required for NbFNR to assist BaMV accumulation, NbFNR/ΔTP, without the transit peptide, was constructed in the prey plasmid. The virus‐encoded polypeptides were cloned into the bait plasmid. The results indicate that NbFNR interacted with the RdRp domain of BaMV replicase (Figure 7a). To validate this interaction, we used a co‐immunoprecipitation assay (Figure 7b). NbFNR/ΔTP‐OFP and BaMV infectious clones carrying the HA‐tagged replicase were co‐expressed in *N. benthamiana* cells. The HA‐tagged replicase could be coprecipitated with NbFNR/ΔTP‐OFP but not with OFP alone (Figure 7b). Therefore, BaMV replicase was transported into the chloroplast and interacted with NbFNR (possibly as part of the replication complex) to regulate viral RNA replication in the chloroplasts.

## 3 DISCUSSION

NbFNR is a flavoenzyme involved in the photosynthesis electron transport chain, catalysing the conversion of NADP^+^ into NADPH. The use of VIGS to reduce the expression of *NbFNR* in leaves and protoplasts of *N. benthamiana* led to the down‐regulation of BaMV accumulation. Further experiments with transiently expressed NbFNR‐OFP or its derivatives indicated that only the expression of functional NbFNR localized in the chloroplast could support BaMV accumulation efficiently.

Most of the chlorosis/mosaic symptoms resulting from virus infection in plants are caused by a change in the expression profile of chloroplast proteins. These proteins are mostly involved in the photosynthetic electron transport chain and the synthesis or degradation of chlorophyll, such as the enzymes in the methylerythritol 4‐phosphate (MEP) pathway (Reinero & Beachy, 1989; Sindelarova et al., 2005). The entry of BaMV CP to chloroplasts can induce symptom development (Lan et al., 2010). The CP of PVX can interact with the transit peptide of chloroplast plastocyanin to induce symptoms (Qiao et al., 2009). The movement protein TGB3 of *Alternanthera mosaic virus* targets the chloroplast and interacts with the photosystem II oxygen‐evolving complex protein PsbO, causing symptom development (Jang et al., 2013).

FNR plays a rate‐limiting step in photosynthesis (Palatnik et al., 2003). The effect is proportional to light intensity and the amount of FNR in chloroplasts. Initially, FNR‐deficient plants show damage to chloroplast membranes, then the level of RuBisCO and other stromal proteins decreases, and finally plants become prone to photo‐oxidative injury (Palatnik et al., 2003). Leaf bleaching is one of the symptoms of FNR‐deficient plants. Therefore, the down‐regulation of *NbFNR* after BaMV inoculation could be related to the chlorosis symptoms appearing on leaves of infected plants (Cheng et al., 2010). The use of rbcL as a loading control for normalization on western blot analysis could lead to erroneous conclusions, as we found previously (Cheng et al., 2010). Because of the role of NbFNR in BaMV accumulation shown in this study, the down‐regulation of NbFNR after BaMV infection could be a strategy for plant defence, lowering the level of a host factor required by the virus and resulting in a reduced accumulation of viral RNA.

Once a positive‐strand RNA virus enters its host cell, it must target an appropriate membrane for replication (Ahlquist et al., 2003; Laliberte & Sanfacon, 2010). *Turnip mosaic virus* (Wei et al., 2010) and *Turnip yellow mosaic virus* (Prod’homme et al., 2003) target the chloroplast envelope membrane, *Potato virus X* (Bamunusinghe et al., 2009), *Tobacco etch virus* (Schaad et al., 1997), *Tobacco mosaic virus* (Kawakami et al., 2004; Nishikiori et al., 2006), *Tomato ringspot virus* (Han & Sanfacon, 2003), and *Cowpea mosaic virus* (Carette et al., 2000) target the endoplasmic reticulum membrane, *Tomato bushy stunt virus* (McCartney et al., 2005) targets the peroxisome, and *Melon necrotic spot carmovirus* (Mochizuki et al., 2009) targets to the mitochondria. Viruses from different families may target the same membrane for replication; conversely, those from the same family may go for different organellar membranes. In the case of BaMV, the nuclear‐encoded chloroplast phosphoglycerate kinase interacts with the genome 3′ UTR and ushers the viral RNA into chloroplasts for replication (Cheng et al., 2013; Lin et al., 2007). Targeting of the chloroplast also probably compromises the parts of the plant defence system associated with the chloroplast (Bhattacharyya & Chakraborty, 2018; Caplan et al., 2008; Serrano et al., 2016; Zhao et al., 2016). However, the entry of viral RNA into chloroplasts could also change structures in the chloroplast, including the thylakoid membranes, where FNR resides, which might interfere with the function of NbFNR and result in chlorosis symptoms after BaMV infection.

Our findings of this study reveal that BaMV replicase interacted with NbFNR (Figure 7) and the function of NbFNR was critical for BaMV accumulation (Figures 4 and 6). When FNR catalyses the conversion of to NADP^+^ to produce NADPH, protons are consumed, which could change the proton concentration in the local microenvironment and might have an effect on BaMV replication. We have demonstrated previously that the initiation of the elongation step of BaMV replication could be regulated by proton concentration (Chen, Tsai, et al., 2017). A high proton concentration favours initiation of BaMV RNA synthesis; conversely, an alkaline condition favours elongation. The chloroplast carbonic anhydrase in *N. benthamiana* enhances the initiation step but not the elongation step in vitro. The results imply that carbonic anhydrase (CO_2_ + H_2_O → HCO_3_^−^ + H^+^, producing protons to create a more acidic condition) and FNR (2Fd_red_ + NADP^+^ + H^+^ → 2Fd_ox_ + NADPH, consuming protons to create a more alkaline condition) could work in as a pair to adjust the proton concentration and regulate BaMV viral RNA synthesis.

In this study, we characterized a gene down‐regulated after BaMV infection, *NbFNR*. The gene product is a nuclear‐encoded chloroplast protein that is essential for photosynthesis. NbFNR in the chloroplast plays a positive regulatory role in BaMV RNA accumulation.

## 4 EXPERIMENTAL PROCEDURES

### 4.1 Plant and viruses

The *N. benthamiana* plants used in this study were grown in a growth room with a constant temperature at 28°C and a 16 h/8 h day/night photoperiod. BaMV was used for inoculation in this study.

### 4.2 Virus particle purification and viral RNA extraction

Approximately 80 g of BaMV‐infected leaves was collected and homogenized with extraction buffer (0.5 M borate pH 9.0, 1 mM EDTA, and 0.1% β‐mercaptoethanol) in a 2‐ml/g ratio. The homogenate was filtered using Miracloth (Millipore Corporation) and centrifuged at 12,000 × *g* for 10 min. K_2_HPO_4_ and CaCl_2_ were added to the supernatant to final concentrations of 1% and 2%, respectively, and the mixture was stirred at 4°C for 10 min. After centrifugation at 12,000 × *g* for 10 min, Triton X‐100 and polyethylene glycol (PEG) 6000 were added to the supernatant to final concentrations of 2% and 6%, respectively, and stirred at 4°C for 30 min. After centrifugation at 12,000 × *g* for 10 min, the viral pellet was suspended in 32 ml BE buffer (50 mM borate pH 8.0, 1 mM EDTA) and layered on top of 5 ml of 20% sucrose. The viral particles were collected after centrifugation at 14,300 × *g* for 1 h and resuspended in 10 ml BE buffer and stored at −80°C.

The viral RNA was extracted from the purified viral particles. The virions were mixed with 1/3 volume of 4× disruption buffer (40 mM Tris‐base, 1 mM EDTA, 0.114% vol/vol acetic acid), 1% sodium dodecyl sulphate (SDS), 1% β‐mercaptoethanol, and 1 mg/ml bentonite, incubated at 65°C for 2–3 min, and extracted with an equal volume of phenol/chloroform. The RNA in the aqueous phase was re‐extracted with phenol/chloroform and precipitated with ethanol. The viral RNA was washed, dried, dissolved in water, and stored at −80°C.

### 4.3 Virus‐induced gene silencing

*N. benthamiana* seedlings approximately 1 month old were used for VIGS. The silencing vector used in this study was previously described (Cheng et al., 2010; Liu et al., 2002). In brief, *Agrobacterium tumefaciens* carrying tobacco rattle virus pTRV1 or pTRV2‐*NbFNR*, ‐*Phytoene desaturase* (PDS), or ‐*luciferase* were cultured at 30°C to OD_600_ = 1 and 5 ml of each were mixed in a 1:1 ratio. Cells were precipitated and resuspended in 10 ml of induction buffer (10 mM MgCl_2_, 10 mM 2‐(*N*‐morpholino)ethanesulfonic acid [MES], 20 μM acetosyringone) and infiltrated into three leaves of each plant. At 2 weeks postinfiltration, the positive control *PDS*‐knockdown plant showed a photobleaching phenotype. Approximately 200 ng of purified BaMV was inoculated on the fourth leaf above the infiltrated leaves.

### 4.4 Protoplast isolation and inoculation of virus RNA

Approximately 2 g of gene‐silenced leaf was digested with 12.5 ml of enzyme solution (0.6 mg/ml pectinase, 12 mg/ml cellulase in 0.1% bovine serum albumin, 0.55 M mannitol‐MES, pH 5.7) at 25°C overnight. The digested cell mixture was filtered through Miracloth. The spun‐down cells were resuspended in 2 ml of 0.55 M mannitol‐MES (pH 5.7) and layered on top of a cushion of 2 ml of 0.55 M sucrose (pH 5.7). Healthy protoplasts were collected from the interphase after centrifugation. After a couple of washes with 0.55 M mannitol‐MES to remove residual sucrose, an aliquot of the collected protoplasts was stained with fluorescein diacetate to examine quality and quantity by fluorescence microscopy. Approximately 5 × 10^5^ protoplasts (around 100 μl) were inoculated with BaMV viral RNA (500 ng) mixed with 150 μl of 40% PEG 6000 (containing 3 mM CaCl_2_) and the protoplasts were resuspended in 1.5 ml of 0.55 M mannitol‐MES. After inoculation, cells were incubated in the 0.55 M mannitol‐MES buffer at 25°C under constant light.

### 4.5 Western blot analysis

Total proteins harvested from inoculated leaves or protoplasts were extracted with 1× Laemmli buffer (2.5 mM Tris‐HCl, pH 8.3; 250 mM glycine; 0.1% SDS), separated on 12% SDS‐polyacrylamide gels and transferred to membranes (PROTRAN BA 85; Schleicher & Schuell). Membranes were incubated with a primary antiserum (rabbit anti‐BaMV coat protein, ‐actin, ‐T7 tag, or ‐OFP), then with secondary antibody (fluorescein‐conjugated affinity‐purified anti‐rabbit IgG) after removal of primary antibodies. Fluorescent signals on the membrane were detected using the LI‐COR Odyssey system (LI‐COR Biosciences).

### 4.6 Total RNA extraction

Approximately 0.2 g of *N. benthamiana* leaf was collected, ground with liquid nitrogen, mixed with 1 ml of Tripure Isolation Reagent (Roche Life Science), and incubated on ice for 5 min. The mixture was shaken vigorously for a few seconds after adding 0.2 ml of chloroform and incubated on ice for 5 min. The aqueous layer containing the RNA was recovered after centrifugation at 4°C with 13,000 × *g* for 15 min. The RNA was precipitated by adding an equal volume of isopropanol, incubated on ice for 30 min, and centrifuged at 4°C with 13,000 × *g* for 10 min. The RNA pellet was washed with 70% ethanol, dried, dissolved in water, and stored at −80°C.

Total RNA from protoplasts was extracted by adding 5× RNA extraction buffer (0.5 M Tris‐HCl, pH 8.0, 50 mM EDTA, 0.5 M NaCl), 0.1% SDS, bentonite 100 mg/ml, and phenol/chloroform with vigorous shaking, incubated on ice for 5 min, and centrifuged at 4°C with 13,000 × *g* for 5 min. About 200 μl of the aqueous layer (containing the RNA) was mixed with 100 μl of 7.5 M ammonium acetate first and then with 750 μl of 100% ethanol. After incubation on ice for 30 min, the RNA was precipitated by centrifugation at 4°C with 13,000 × *g* for 25 min.

### 4.7 Reverse transcription quantitative PCR

Approximately 1 µg (4 µl) of total RNA after treatment with DNase I and 1 µl of oligo(dT)_15_ primer (20 pmol/µl) were mixed, incubated at 70°C for 5 min, and placed on ice. The mixture was added to 15 µl of reagents (6.1 µl double‐deionized water, 4 µl of 5× buffer, 2.4 µl of 25 mM MgCl_2_, 1 µl of 10 mM dNTP, 0.5 µl of RNase inhibitor, and 1 µl reverse transcriptase) for reverse transcription at 42°C for 1 h. The cDNA (3 μl) of *NbFNR* and *actin* was PCR‐amplified using Master Mix (2×) containing all the components, including SYBR Green I (KAPA Biosystems), with 0.2 µM specific primer pairs for *NbFNR*, NbFNR/qPCR5′ (5′‐GCAGTTTCTCTTCCATCATCCAAGTCC‐3′) and NbFNR/qPCR3′ (5′‐CTCTGTGGTCACCTGGGCTCTG‐3′), and for *actin*, β‐actin3′ (5′‐GTGGTTTCATGAATGCCAGCA‐3′), and β‐actin5′ (5′‐GATGAAGATACTCACAGAAAGA‐3′). Real‐time quantitative PCR was performed in a thermocycler (TOptical Gradient 96; Biometra). The reaction without a template or reverse transcriptase was a negative control, and *actin* was measured for normalization. All samples were run at least three times.

### 4.8 Northern blot analysis

Total RNA (2.8 μl) isolated from inoculated leaves or protoplasts was mixed with 1.2 µl of 10× sodium phosphate buffer (100 mM, pH 7.0), 6 µl dimethyl sulphoxide (DMSO), 2 µl of 6 M glyoxal and water to a final 12 µl of reaction mixture. The reaction mixture was incubated at 50°C for 1 h, electrophoresed on a 1% agarose gel prepared with 10 mM phosphate buffer, and transferred to a membrane (Hybond‐N^+^; GE Healthcare) as described (Weiland & Dreher, 1989). The membrane was incubated with 10 ml of hybridization buffer (prepared by mixing 0.75 ml of 20× SET [3 M NaCl, 40 mM EDTA, 0.6 M Tris‐HCl pH 8.0], 0.9 ml of 10% SDS, 3 ml of 50× Denhardt's solution, 150 µl of yeast total RNA [25 µg/µl] and 10.2 ml of water) at 65°C for at least 1 h, prehybridization buffer was removed, and a 10 million cpm probe was added into 5 ml of buffer for rotating overnight. The hybridization probes were ^32^P‐labelled 0.6‐kb and 0.7‐kb RNA transcripts complementary to the 3′‐end of plus‐strand and minus‐strand BaMV viral RNA, respectively (Chen et al., 2005; Huang & Tsai, 1998). After hybridization, the membrane was washed with washing buffer (0.5× SET, 0.1% SDS, 0.1% sodium pyrophosphate). The radioactive signals were detected and quantified by using a phosphorimager (Fujifilm BAS 1500).

### 4.9 Transient expression and localization of NbFNR

To obtain the full‐length *NbFNR* cDNA, the open reading frame of *NbFNR* was amplified by reverse transcription PCR with the NbFNR5′ primer (5′‐TCTAGAATGGCTGCTGCAGTAAGTGCTG‐3′; *Xba*I site underlined) and NbFNR3′ primer (5′‐GGTACCACCCATTTGCTGTCCACCAGTCATGCTAGCC ATGTATACTTCAACATTCC‐3′; *Kpn*I site underlined). The amplified *NbFNR* cDNA fragment was cloned into the pGEM‐T Easy vector (Promega) and verified by sequencing. The full‐length cDNA was then subcloned into the pEpyon binary vector, which carries the *mOrange2* reporter gene (Shaner et al., 2008), with the restriction enzyme sites *Xba*I and *Kpn*I. A mutant without the transit peptide, NbFNR/ΔTP, was constructed similarly using the primer set NbFNR/ΔTP‐5′ (5′‐GTCTAGAATGGCCCAGGTGACCACAGA‐3′; *Xba*I site underlined) and NbFNR3′ for PCR. The functional activity mutant (S140A/E360A) (Dumit et al., 2010), NbFNR/mut, was constructed by ligating the vector overlapped with two fragments (m1 and m2) according to the NEBuilder HiFi DNA assembly cloning kit (New England Biolabs). The 5′ insert fragment (m1) was amplified with the primer set NbFNR/m1‐5′ (5′‐*ATGGGCGGCCGCGGGAATTCGAT* TCTAGAATGGCTACTGCAGTAAGTGCT‐3′; T‐vector sequence in italics and *Xba*I site underlined) and NbFNR/m1‐3′ (5′‐GCTAGCAATGGCGTATAATCTGAGCT‐3′). The 3′ fragment (m2) was amplified with the primer set NbFNR/m2‐5′ (5′‐GCTCAGATTATACGCCATTGCTAGCAGTGC‐3′) and NbFNR/m2‐3′ (5′‐*GCCGCGAATTCACTAGTGAT* GGTACC **ACCCATTTGCTGTCC ACCAGTCATGCTAGCCAT**GTAGACTGCAACATTCCATTGCTCTG‐3′; T‐vector sequence in italics; *Kpn*I site underlined; T7‐tag sequence in bold).

Approximately 3‐week‐old *N. benthamiana* plants were used for the transient expression experiments. The plasmids containing the full‐length cDNA of *NbFNR* and its derivatives fused with a T7‐tag or OFP were transformed into *A. tumefaciens* C58C1 by electroporation. *Agrobacterium* containing these constructs was co‐infiltrated with a silencing suppressor (HC‐Pro) into a single leaf of each *N. benthamiana*, and 12 h later 200 ng of BaMV virions was mechanically inoculated onto the infiltrated leaf. Total protein of the inoculated leaf was harvested at 3 days postinoculation (dpi). The expression of *NbFNR* and its derivatives and the accumulation of BaMV CP were examined by western blot analysis.

To examine the localization of NbFNR and its derivatives, *Agrobacterium* containing pNbFNR‐OFP or its derivatives was mixed with that containing a silencing suppressor (HC‐Pro) in a 1:1 ratio and infiltrated into *N. benthamiana* leaves. The fluorescent signal emitted by NbFNR‐OFP was examined at 3 dpi by confocal laser scanning microscopy (FV1000; Olympus).

### 4.10 Replicase complex preparation and in vitro replication assay

BaMV‐ or PVX‐infected leaves were collected at 5 dpi and homogenized with extraction buffer (50 mM Tris‐HCl pH 7.6, 15 mM MgCl_2_, 120 mM KCl, 0.1% β‐mercaptoethanol, 20% glycerol, 1× protease inhibitor cocktail [Roche]) in 2 ml/g of buffer/leaf ratio. The homogenate was filtered through Miracloth and centrifuged at 500 × *g* for 10 min. The pellet was resuspended in suspension buffer (50 mM Tris‐HCl pH 8.2, 10 mM MgCl_2_, 1 mM dithiothreitol [DTT], 1× protease inhibitor cocktail) after centrifugation at 30,000 × *g* for 35 min. Approximately 2 ml of the extract was loaded on a 28 ml 20%–60% continuous gradient of sucrose with gradient buffer (50 mM Tris‐HCl pH 8.0, 10 mM NaCl, 1 mM EDTA, 5% glycerol, 1× protease inhibitor cocktail, and 1 mM DTT) and centrifuged at 72,100 × *g* for 7.2 h. The three fractions (3 ml each fraction) with the highest RdRp activity (the endogenous template activity assay) were pooled and stirred with 1.5% NP‐40 for 1 h to solubilize the membrane‐associated RdRp.

For the in vitro replication assay with the endogenous RNA templates, 8 µl of the replicase complex preparation was added to 50 µl of reaction containing 50 mM Tris‐HCl pH 8.2, 2 mM (A, C, and G)TP, 2 µM UTP, 0.066 µM [α‐^32^P]UTP (3000 Ci mmol/L; Dupont‐NEN), 3 mM MgCl_2_, 10 mM DTT, 8 mg bentonite, and 12 U RNase OUT (Invitrogen) with various concentrations of heparin (Sigma‐Aldrich) at 30°C for 1 h. The reaction was stopped by adding 150 µl of 5 mM EDTA, extracted with phenol/chloroform, and precipitated with ethanol. The radioactive RNA products were resolved on a 1% agarose gel and quantified by using the PhosphoImaging analyzer Amersham Typhoon (GE Healthcare Life).

### 4.11 Yeast two‐hybrid assay

The yeast Hybrid Hunter system (Invitrogen) was used for the interaction between bait and prey protein that could activate the expression of the reporter genes (*his3* and *lacZ* ) in *Saccharomyces cerevisiae* L40. The gene fragment encoding NbFNR/ΔTP was inserted into the prey plasmid pYESTrp2, designated pYES‐FNR/ΔTP. The virus‐encoded genes were inserted into the bait plasmid pHybLex/Zeo and designated pLEX/capping, ‐helicase, ‐RdRp, ‐ TGBp1, ‐TGBp2, ‐TGBp3, and ‐CP. The bait plasmids and prey plasmid were co‐transformed into *S. cerevisiae* L40 and selected on Trp−/His−/Zeocin plates.

### 4.12 Immunoprecipitation assay

*Agrobacterium* cultures containing pBIN/OFP or ‐/NbFNR/ΔTP‐OFP were mixed with those containing pBIN/HC‐Pro and pKB/Rep‐HA (the full‐length of BaMV infectious cDNA with HA‐tagged replicase) in a 1:1:1 ratio and infiltrated into the leaves of *N. benthamiana* plants. Total protein was extracted from the leaves after 3 days with extraction buffer (20 mM Tris‐HCl, pH 7.5, 2 mM MgCl_2_, 300 mM NaCl, 0.5% NP‐40, 5 mM DTT, and 1× EDTA‐free protease inhibitor [Roche]) and purified by using anti‐OFP magnetic beads (RFP‐Trap_M; ChromoTek). The beads were then washed with binding buffer (20 mM Tris‐HCl, pH 7.5, 2 mM MgCl_2_, 300 mM NaCl) and Tris‐buffered saline. The proteins pulled down were analysed by western blot analysis with anti‐HA (Sigma‐Aldrich) or anti‐OFP (Yao‐Hong Biotechnology) antibodies.

## COMPETING INTERESTS

The authors declare no competing financial or nonfinancial interests.

## AUTHOR CONTRIBUTIONS

I.H.C., X.Y.C., G.Z.C., and Y.P.H. performed the experiments. I.H.C., Y.H.H., and C.H.T. took part in data analysis. I.H.C. and C.H.T. designated the research and wrote the article.

## Supporting information


**FIGURE S1** Sequence alignment. The amino acid sequences of ferredoxin NADP^+^ oxidoreductase derived from *Nicotiana tabacum* (GenBank accession no. O04977.1) and *N. benthamiana* (GenBank accession no. MH511674) were aligned

**FIGURE S2** The accumulation of BaMV coat protein in *NbFNR*‐knockdown plants. CP, BaMV coat protein; rbcL, RuBisCO large subunit and used as a loading control for normalization. Data above bars are mean ± *SE*. Asterisks indicate statistically significant differences by Student’s *t* test (***p < 0.001)

**FIGURE S3** The accumulation of BaMV coat protein in plants transiently expressing NbFNR. (a) Western blot analysis of the expression of OFP‐T7 and NbFNR‐T7 in agroinfiltrated plants. (b) Western blot analysis of BaMV coat protein level in plants transiently expressing NbFNR. Total protein was extracted from plants transiently expressing OFP‐T7 or NbFNR‐T7 at 3 days postinoculation with 200 ng of BaMV virions. OFP‐T7, OFP with T7‐tag transiently expressed plants; FNR‐T7, NbFNR with T7‐tag transiently expressing plants; CP, coat protein; rbcL, RuBisCO large subunit was a loading control. Data above bars are mean ± *SE* obtained from three independent experiments. Asterisks indicate statistically significant differences by Student’s *t* test (***p < 0.001)

**FIGURE S4** Localization of *NbFNR‐OFP* in *Nicotiana benthamiana* leaves. Localization of transiently expressed OFP only and NbFNR‐OFP in *N. benthamiana* leaves detected by confocal microscopy. OFP is in green and the autofluorescence of chloroplasts is in red. Scale bar: 10 μm

**FIGURE S5** Localization of *NbFNR‐OFP* in *Nicotiana benthamiana* leaves after BaMV infection. (a) Localization of transiently expressed NbFNR‐OFP in *N. benthamiana* leaves and with the presence of BaMV viral vector (CBG) expressing green fluorescent protein (GFP) detected by confocal microscopy. (b) The enlargement of the merge panel from (a) without the GFP channel. GFP is in green, OFP is in red, and the autofluorescence of chloroplasts is in blue. Scale bar: 10 μm

## Data Availability

The data supporting the findings of this study are available from the corresponding author upon request.
