# Supplementary material for: The function of chloroplast ferredoxin‐NADP+ oxidoreductase positively regulates the accumulation of bamboo mosaic virus in Nicotiana benthamiana
Source: Mol Plant Pathol. 2021 Dec 17;23(4):503–15. doi: 10.1111/mpp.13174 (PMC8916203; doi:10.1111/mpp.13174)
Supplement: Supplementary file 1 — FIGURE S1 Sequence alignment. The amino acid sequences of ferredoxin NADP+ oxidoreductase derived from Nicotiana tabacum (GenBank accession no. O04977.1) and N. benthamiana (GenBank accession no. MH511674) were aligned [file MPP-23-503-s005.pdf]

```
       1                                                                                  86
NbFNR  MATAVSAAVSLPSSKSTSFPTRTSIISPEKINFNKVPLYYRNVSAGSRVASIRAQVTTEAPAKVEKISKKQDEGVVVNKFRPKEPY
NtFNR  MATAVSAAVSLPSSKSTSFSSRTSIISTDKINFNKVPLYYRNVSGGSRLVSIRAQVTTEAPAKVEKISKKQDEGVIVNKFRPKEPY

       87                                                                                172
NbFNR  IGRCLLNTKITGDDAPGETWHMVFSTEGEVPYREGQSIGVIADGVDANGKPHKLRLYSIASSALGDFGDSKTVSLCVKRLIYTNDK
NtFNR  VGRCLLNTKITGDDAPGETWHMVFSTEGEVPYREGQSIGVIADGVDANGKPHKLRLYSTASSALGDFGDSKTVSLCVKRLVYTNDK

       173                                                                               258
NbFNR  GEEVKGVCSNFLCDLKPGAEVKITGPVGKEMLMPKDPNATIVMLATGTGIAPFRSFLWKMFFEKHEDYKFNGLAWLFLGVPTSSSL
NtFNR  GEEVKGVCSNFLCDLKPGAEVKITGPVGKEMLMPKDPNATVIMLATGTGIAPFRSFLWKMFFEKHEDYKFNGTAWLFLGVPTSSSL

       259                                                                               344
NbFNR  LYKEEFEKMKEKAPENFRLDFAVSREQTNEKGEKMYIQTRMAQYAEELWGLLQKDNTFVYMCGLKGMESGIDDIMTSLAARDGIVW
NtFNR  LYKEEFEKMKEKAPENFRLDFAVSREQTNEKGEKMYIQTRMAQYAEELWTLLQKDNTFIYMCGLKGMEQGIDEIMSALAERDGIVW

       345              362
NbFNR  ADYKKQLKKAEQWNVEVY
NtFNR  ADYKKQLKKAEQWNVEVY
```

FIGURE S1 Sequence alignment. The amino acid sequences of ferredoxin NADP$^+$ oxidoreductase derived from *Nicotiana tabacum* (GenBank accession no.: O04977.1) and *N. benthamiana* (GenBank accession no.: MH511674) were aligned.
