# Supplementary material for: The function of chloroplast ferredoxin‐NADP+ oxidoreductase positively regulates the accumulation of bamboo mosaic virus in Nicotiana benthamiana
Source: Mol Plant Pathol. 2021 Dec 17;23(4):503–15. doi: 10.1111/mpp.13174 (PMC8916203; doi:10.1111/mpp.13174)
Supplement: Supplementary file 2 — FIGURE S2 The accumulation of BaMV coat protein in NbFNR‐knockdown plants. CP, BaMV coat protein; rbcL, RuBisCO large subunit and used as a loading control for normalization. Data above bars are mean ± SE. Asterisks indicate statistically significant differences by Student’s t test (***p < 0.001) [file MPP-23-503-s001.pdf]

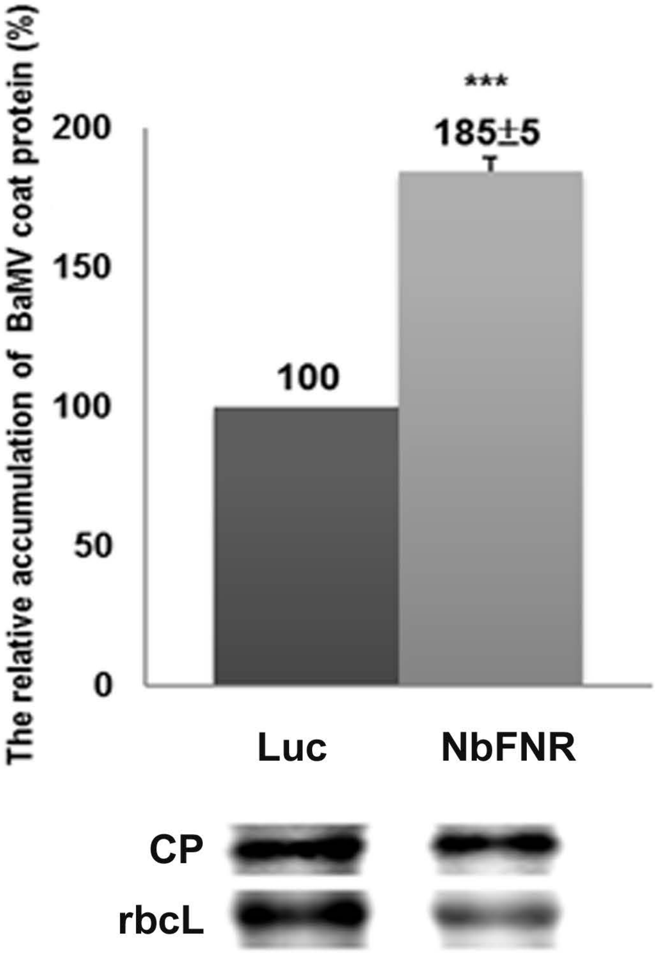

FIGURE S2 The accumulation of BaMV coat protein in *NbFNR*-knockdown plants. CP: BaMV coat protein; rbcL: RuBisCo large subunit and used as a loading control for normalization. Data above bars are mean±SEM. Asterisks indicate statistically significant differences by Student *t* test (***, $p<0.001$).
