# Supplementary material for: The function of chloroplast ferredoxin‐NADP+ oxidoreductase positively regulates the accumulation of bamboo mosaic virus in Nicotiana benthamiana
Source: Mol Plant Pathol. 2021 Dec 17;23(4):503–15. doi: 10.1111/mpp.13174 (PMC8916203; doi:10.1111/mpp.13174)
Supplement: Supplementary file 3 — FIGURE S3 The accumulation of BaMV coat protein in plants transiently expressing NbFNR. (a) Western blot analysis of the expression of OFP‐T7 and NbFNR‐T7 in agroinfiltrated plants. (b) Western blot analysis of BaMV coat protein level in plants transiently expressing NbFNR. Total protein was extracted from plants transiently expressing OFP‐T7 or NbFNR‐T7 at 3 days postinoculation with 200 ng of BaMV virions. OFP‐T7, OFP with T7‐tag transiently expressed plants; FNR‐T7, NbFNR with T7‐tag transiently expressing plants; CP, coat protein; rbcL, RuBisCO large subunit was a loading control. Data above bars are mean ± SE obtained from three independent experiments. Asterisks indicate statistically significant differences by Student’s t test (***p < 0.001) [file MPP-23-503-s003.pdf]

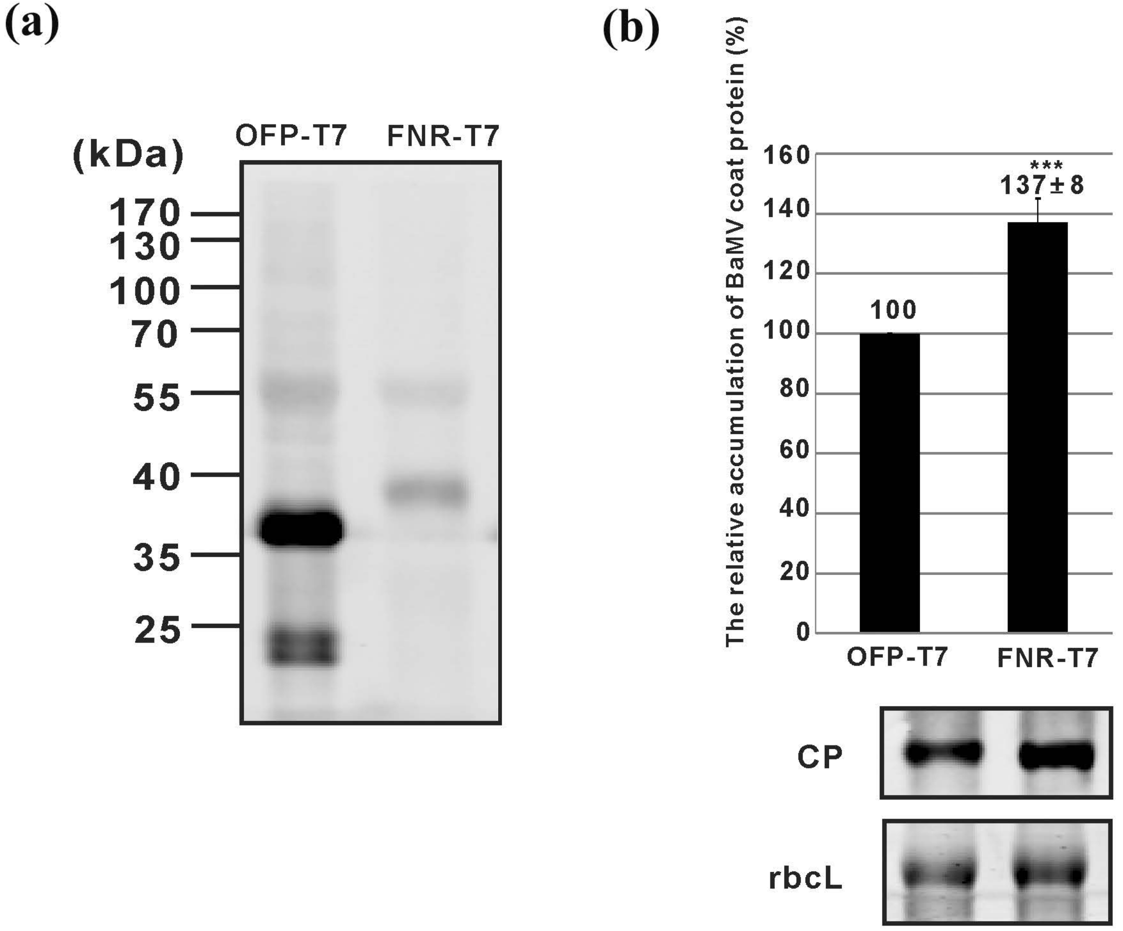

FIGURE S3 The accumulation of BaMV coat protein in plants transiently expressing NbFNR. (a) Western blot analysis of the expression of OFP-T7 and NbFNR-T7 in agroinfiltrated plants. (b) Western blot analysis of BaMV coat protein level in plants transiently expressing NbFNR. Total protein was extracted from plants transiently expressing OFP-T7 or NbFNR-T7 at 3 days post-inoculation with 200 ng of BaMV virions. OFP-T7: OFP with T7-tag transiently expressed plants; FNR-T7: NbFNR with T7-tag transiently expressed plants; CP: coat protein; rbcL: RuBisCO large subunit was a loading control. Data above bars are mean±SE obtained from three independent experiments. Asterisks indicate statistically significant differences by Student *t* test (***, $p<0.001$).
