# Supplementary material for: The function of chloroplast ferredoxin‐NADP+ oxidoreductase positively regulates the accumulation of bamboo mosaic virus in Nicotiana benthamiana
Source: Mol Plant Pathol. 2021 Dec 17;23(4):503–15. doi: 10.1111/mpp.13174 (PMC8916203; doi:10.1111/mpp.13174)
Supplement: Supplementary file 4 — FIGURE S4 Localization of NbFNR‐OFP in Nicotiana benthamiana leaves. Localization of transiently expressed OFP only and NbFNR‐OFP in N. benthamiana leaves detected by confocal microscopy. OFP is in green and the autofluorescence of chloroplasts is in red. Scale bar: 10 μm [file MPP-23-503-s002.pdf]

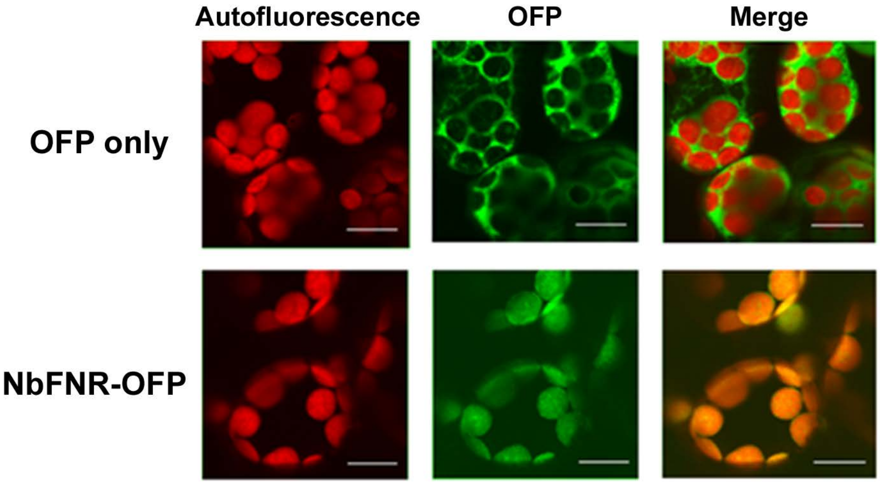

FIGURE S4 Localization of *NbFNR-OFP* in *N. benthamiana* leaves. Localization of transiently expressed OFP only and NbFNR-OFP in *N. benthamiana* leaves detected by confocal microscopy. OFP is in green, and the autofluorescence of chloroplasts is in red. Scale bar: 10 μm.
