# Supplementary material for: The function of chloroplast ferredoxin‐NADP+ oxidoreductase positively regulates the accumulation of bamboo mosaic virus in Nicotiana benthamiana
Source: Mol Plant Pathol. 2021 Dec 17;23(4):503–15. doi: 10.1111/mpp.13174 (PMC8916203; doi:10.1111/mpp.13174)
Supplement: Supplementary file 5 — FIGURE S5 Localization of NbFNR‐OFP in Nicotiana benthamiana leaves after BaMV infection. (a) Localization of transiently expressed NbFNR‐OFP in N. benthamiana leaves and with the presence of BaMV viral vector (CBG) expressing green fluorescent protein (GFP) detected by confocal microscopy. (b) The enlargement of the merge panel from (a) without the GFP channel. GFP is in green, OFP is in red, and the autofluorescence of chloroplasts is in blue. Scale bar: 10 μm [file MPP-23-503-s004.pdf]

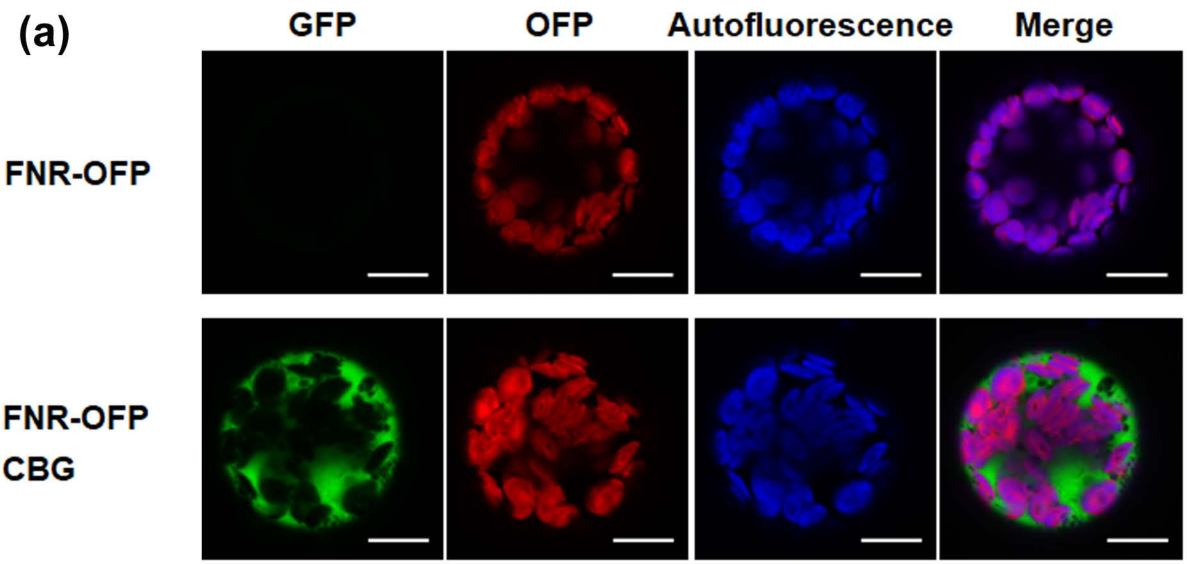


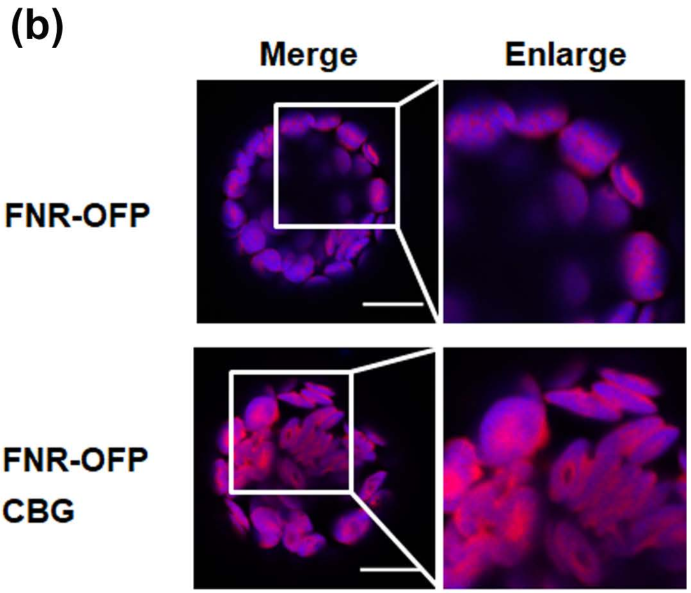


**FIGURE S5** Localization of *NbFNR-OFP* in *N. benthamiana* leaves after BaMV infection. (a) Localization of transiently expressed NbFNR-OFP in *N. benthamiana* leaves and with the presence of BaMV viral vector (CBG) expressing green fluorescent protein (GFP) detected by confocal microscopy. (b) The enlargement of the merge panel from (a) without the GFP channel. GFP is in green, OFP is in red, and the autofluorescence of chloroplasts is in blue. Scale bar: 10 μm.
